# The INFINITY study protocol: a retrospective cohort study on decision making and clinical impact of biomarker-driven precision oncology in routine clinical practice

**DOI:** 10.1186/s12885-023-11046-3

**Published:** 2023-06-13

**Authors:** Uwe M. Martens, Jan Schröder, Fee Bengsch, Ludger Sellmann, Sabine Busies, Stefanie Frank-Gleich, Matthias Zaiss, Thomas Decker, Andreas Schneeweiss, Martin Schuler, Sina Grebhardt, Stefan Zacharias, Norbert Marschner, Benjamin Kasenda, Karin Potthoff, Corinne Vannier

**Affiliations:** 1 Clinic for Hematology, Oncology and Palliative Care, SLK Kliniken Heilbronn, Heilbronn, Germany; 2 MOLIT Institute for Personalized Medicine, Heilbronn, Germany; 3 Practice for Hematology and Internal Oncology, Mülheim a.d.R, Germany; 4 iOMEDICO, Ellen-Gottlieb-Straße 19, 79106 Freiburg im Breisgau, Germany; 5 Practice for Oncology, Mönchengladbach, Germany; 6 Joint Practice for Internal Medicine, Hematology, Oncology, Gastroenterology, Halle (Saale), Germany; 7 Center for Interdisciplinary Oncology & Hematology, Freiburg, Germany; 8 Study Center for Oncology Ravensburg, Ravensburg, Germany; 9 National Center for Tumor Diseases, Heidelberg University Hospital, Heidelberg, Germany; 10 German Cancer Research Center, Heidelberg, Germany; 11 West German Cancer Center, Department of Medical Oncology, University Hospital Essen, University Duisburg-Essen, Essen, Germany; 12 Medical Oncology, University Hospital and University of Basel, Basel, Switzerland

**Keywords:** Precision oncology, Non-standard targeted therapy, Actionable alteration, Real-world data, Non-interventional study

## Abstract

**Background:**

Precision oncology, defined as treatment of patients with targeted therapies matched to specific molecular alterations, has entered routine clinical practice. Particularly in patients with advanced cancer or hematologic malignancies, for whom no further standard therapies are available, this approach is increasingly applied as last resort option outside of the approved indication. However, data on patient outcomes are not systematically collected, analyzed, reported, and shared. We have initiated the INFINITY registry to provide evidence from routine clinical practice to fill this knowledge gap.

**Methods:**

INFINITY is a retrospective, non-interventional cohort study conducted at approximately 100 sites in Germany (office-based oncologists/hematologists and hospitals). We aim to include 500 patients with advanced solid tumors or hematologic malignancies who received a non-standard targeted therapy based on potentially actionable molecular alterations or biomarkers. INFINITY aims to provide insights into the use of precision oncology in routine clinical practice within Germany. We systematically collect details on patient and disease characteristics, molecular testing, clinical decision-making, treatment, and outcome.

**Discussion:**

INFINITY will provide evidence on the current biomarker landscape driving treatment decisions in routine clinical care. It will also provide insights on effectiveness of precision oncology approaches in general, and of specific drug class/alteration matches used outside their approved indications.

**Trial registration:**

The study is registered at ClinicalTrials.gov, NCT04389541.

## Background

Oncology is currently experiencing a paradigm shift from a “one-drug-fits-all” treatment model towards a tailored, molecular targeted or biomarker-driven treatment approach [1–4]. The goal is to choose the most effective, best tolerated drug for the patient, based on the molecular profile of the individual disease.

To investigate the efficacy of this novel treatment approach, new clinical trial concepts have evolved, shifting from a tumor type focus to a molecular profile focus. Thus, biomarker-guided trial designs have recently emerged, such as basket trials allocating patients to matched treatments based on molecular alteration across cancer types, or umbrella trials involving one cancer type and different treatments matched to genomic alterations [2, 5].

Several precision oncology trials have shown promising results, suggesting that molecular-matched targeted therapy has the potential to improve outcomes compared to conventional therapy [2, 6–11]. However, it is difficult to conduct biomarker-driven clinical trials and thus, reliable evidence from randomized trials is still lacking. In this context, real-world data are a valuable data source with the potential to address and couple real-world molecular and clinical data and to provide deep insights also into sequential treatment decisions and overall outcomes.

The number of available targeted drugs is steadily increasing [12, 13]. First drugs have been approved in a tumor-agnostic manner based on the presence of molecular alterations instead of specific tumor types: In 2017, the Food and Drug Administration (FDA) granted the first tumor-agnostic approval for pembrolizumab for patients with unresectable or metastatic, microsatellite instability-high or mismatch repair deficient (MSI-H/dMMR) solid tumors. As of 2021, four tumor-agnostic FDA approvals have been granted for three drugs (pembrolizumab, larotrectinib and entrectinib) based on three molecular alterations (MSI-H/dMMR, *neurotrophic tyrosine receptor kinase* (*NTRK*) gene fusion and high tumor mutational burden (TMB-H)) [14]. Furthermore, a growing number of targeted therapies has been approved in more than one tumor type based on specific molecular alterations (e.g., BRAF, HER2/ERBB2, RET, BRCA1/2, PD-L1) including the approval of dabrafenib/trametinib for pediatric patients with BRAF V600E mutant glioma and selpercatinib, a selective kinase inhibitor for patients with rearranged during transfection (RET) fusion-positive tumor by 2023 [5, 15, 16].

Besides conventional diagnostic techniques including polymerase chain reaction (PCR), immunohistochemistry (IHC), (fluorescent) in situ hybridization ((F)ISH), reverse transcription PCR (RT-PCR) and microarrays, next generation sequencing (NGS) techniques have transformed the diagnostic field and are now broadly available [5, 11].

Increasing availability and use of NGS testing as well as targeted therapies nourish the hope that these new technologies will quickly translate into patient clinical benefits, especially in situations where no further standard treatment is available. However, clinical outcomes of this approach are not systematically collected, analyzed, and reported.

The currently available evidence on the benefit of molecular-matched therapies used outside of their approved indication still carries substantial uncertainties. The only randomized trial in the field (SHIVA) found no clear benefit for patients receiving molecular-matched treatments used outside their labeled indications compared to standard of care (PFS 2.3 vs. 2.0 months) [17]. However, this trial was conducted several years ago, and diagnostic techniques and availability of targeted therapies have improved since then. MyPathway, a multicenter, multiple basket phase IIa study, evaluates efficacy of treatments targeting molecular alterations of HER2, epidermal growth factor receptor (EGFR), BRAF and the Hedgehog pathway in subprotocols [18]. The TAPUR phase II multi-basket trial, investigating safety and efficacy of commercially available targeted antineoplastic drugs in patients with metastatic solid tumors harboring potentially actionable molecular alterations, was initiated in 2016 [19]. While proof for a clinical benefit is missing in some patient cohorts, encouraging results in other cohorts [20] provide evidence of the feasibility and potential value of the basket study design in precision medicine [18, 21, 22]. The TAPUR trial is still ongoing, but first study results already helped to further shape the field: Treatment of metastatic colorectal cancer patients with *FMS-like tyrosine kinase-3 (FLT-3)* amplification with sunitinib was found to be ineffective; while on the other hand pembrolizumab monotherapy was reported to have anti-tumor activity in metastatic breast cancer patients with TMB-H, supporting the recent FDA approval [21, 22].

The INFINITY registry will provide systematically collected real-world data on the use of molecular-matched targeted therapies outside of their approved indication for treatment of patients with advanced solid tumors or hematologic malignancies, for whom no standard therapy is available. INFINITY explores how precision oncology is applied in routine clinical practice in Germany, how decisions are made by the treating physicians, which diagnostic tests are utilized to identify potentially actionable alterations, which substance classes of targeted therapies are used, and which molecular alterations are targeted. Effectiveness of matched therapies in the total patient cohort and in patient subgroups will be analyzed, aiming to generate hypotheses on effective or ineffective drug class/alteration matches. Furthermore, a decentral tissue sample biobank is set up, collecting sample information for genomic analysis for future translational research projects.

## Methods/design

### Objectives

INFINITY collects real-world data on precision oncology in Germany with the objective to assess treatment and outcomes of adult patients with advanced solid tumors or hematologic malignancies who received a non-standard targeted therapy (NSTT; i.e., a targeted therapy used outside the labeled indication and not recommended in guidelines) based on an actionable molecular alteration or associated with biomarker(s). NSTT administered in this study will be matched to targetable genomic or proteomic alterations or matched to biomarkers (i.e. biomarker-driven precision immunotherapy / checkpoint inhibitor / targeted therapy based on biomarker(s) associated with treatment benefit also including (but not restricted to) upstream and/or downstream intracellular targets). In particular, data on patient and disease characteristics, molecular testing, physician´s decision making, treatment (assessed as substance class), and outcome will be evaluated.

### Study design

INFINITY is a retrospective, multicenter, non-interventional study, conducted at approximately 100 study sites in Germany, including hospitals and office-based oncologists. 500 patients will be enrolled over a recruitment period of 3 years, which started in April 2020.

### Eligibility criteria

Inclusion criteria:

- Patients with advanced solid tumors (i.e., locally advanced, inoperable and/or metastatic) or hematologic malignancies not eligible for standard therapy options (i.e., without further treatment options with drugs approved for the specific indication based on the judgement of the treating physician)
- Started or completed a non-standard targeted therapy based on a potentially actionable alteration or biomarker identified by molecular diagnostics
- Availability of molecular diagnostic test results that informed clinical decision to apply the non-standard targeted therapy
- Targeted therapy must be non-standard at time point of patient enrollment/registration
- Age ≥ 18 years
- Signed and dated informed consent form (only if patient is alive at time of data entry into the project, not applicable for inclusion of deceased patients’ data)

Exclusion criteria:

- The non-standard targeted therapy was administered within a clinical trial
- The targeted therapy is non-standard because it was given in another therapy line, without a designated prior therapy or in combination with a different chemotherapy backbone, as specified by summary of product characteristics and/or treatment guidelines.

### Data collection

Study sites are asked to retrospectively document all patients who meet the eligibility criteria (including deceased patients) until 2023.

Clinical routine data documented by study sites is structured and transferred by the site staff into a secure web-based eCRF system (iostudy office). The iostudy office edc system is a fully validated secure software that conforms to 21 CRF Part 11 requirements. As source of documentation, routine medical records are used. Sites will document data regarding patient and disease characteristics (e.g. sex, entity, staging, histology) as well as treatment details of NSTT and prior and subsequent therapies applied. The investigator’s treatment decision process is evaluated using a paper-based questionnaire comprising questions on factors influencing treatment decisions as well as information on respective potentially actionable alteration including ESCAT levels, which are transferred into the eCRF. eCRF documentation will be checked for plausibility and completeness both centrally and on-site by data managers, medical managers, and clinical monitors, respectively.

Study sites send pseudonymized diagnostic reports (e.g. pathology reports, reports on sequencing results such as NGS) and molecular tumor board (MTB) reports, if applicable, including treatment recommendations to iOMEDICO. Dedicated and trained iOMEDICO staff extracts information from the reports and enters data on all reported immunohistochemistry or mutational profiling as well as molecular alterations including complex sequencing results (including test panels and further information) into a pre-specified electronic database. These data are documented as described in the report but in analogy with the HGVS (Human genome variation society) code for proteomic und genomic sequence variants [23]. Independent checks for consistency of data entries by a second reviewer are performed. This is to ensure central quality control for these comprehensive and complex data. This structured data capture enables standardized and structured analyses.

Additionally, we have established a decentral tissue sample biobank where information on tumor samples including contact details of local pathology departments is captured. This enables us to request samples for upcoming translational research projects.

### Descriptive analyses

Regarding patient and disease characteristics as well as therapies applied, the following data will be analyzed:

- Patient and disease characteristics including e.g., sex, body mass index (BMI), Eastern Cooperative Oncology Group (ECOG) performance status, type of health insurance, comorbidities and tumors types, time from primary diagnosis to start of first NSTT, age at start of first NSTT
- Details on NSTT including substance classes, treatment duration as well as prior and subsequent antineoplastic therapy lines

Characteristics regarding diagnostics and identified molecular alterations include the following:

- Frequencies: type of molecular diagnostic testing performed, proteins and genes tested, multigene panels or NGS in molecular diagnostics, type (by panel size) of NGS library sequenced, altered proteins and genes, if tested, treatment recommendations given in molecular diagnostic reports, implemented treatment recommendations given in molecular diagnostic reports, use of a MTB, implementation of the treatment recommendation given by MTB
- Clinical decision making: Frequency of answers rated with a Likert scale on patient’s non-suitability for standard therapy options / choice of performed molecular diagnostics / choice of selected molecular target and targeted non-standard therapy / reasons for the selection of targeted non-standard therapy / primary goal of the non-standard targeted therapy / expected advantages of the non-standard targeted therapy and frequency of ESMO Scale of Clinical Actionability for molecular Targets (ESCAT) evidence levels
- Turnaround times:
  - ◦ Duration from sample receipt to molecular testing result
  - ◦ Duration from molecular testing result to start of NSTT

### Effectiveness outcomes

Effectiveness of non-standard targeted therapy will be analyzed as follows:

- Overall survival (OS)
- Progression-free survival (PFS)
- PFS ratio (i.e., PFS of non-standard targeted therapy divided by PFS of preceding therapy)
- Time to treatment failure (TTF)
- TTF ratio (i.e., TTF of non-standard targeted therapy divided by TTF of preceding therapy)
- Tumor response: Best overall response (BOR); overall response rate (ORR); disease control rate (DCR); Time to Response (TTR); Duration of Response (DOR)

### Statistical analysis

No formal sample size calculation was performed. The sample size of 500 patients has been chosen to enable subgroup analyses for e.g., specific tumor entities with a predefined minimum number of patients. For example, given the distribution of patients for specific tumor entities from the MOSCATO trial [8], we expect 50 patients or more in each of the following cancer entities: head and neck cancer, gastrointestinal cancer, lung cancer, breast cancer und genitourinary cancer. Furthermore, given 100 recruiting sites with 1-2 eligible patients per year and 4 documentation periods, it can reasonably be expected to reach the sample size of 500 patients in time.

All patients fulfilling the eligibility criteria and with documented start date of NSTT will be included in the full analysis set (FAS). All variables will be analyzed in a descriptive manner. Categorical variables will be presented as absolute and relative frequencies and continuous variables as number of observations, median and range. Time-to-event variables (PFS and OS) will be analyzed using the Kaplan-Meier method. PFS is defined as the time from start of therapy to date of documented tumor relapse/progression or death due to any cause, whichever comes first. OS is defined as the time from start of therapy to date of death due to any cause. Patients with no event will be censored at the date of last contact or start of subsequent systemic therapy. The PFS ratio will be calculated to compare PFS of NSTT with time to progression (TTP) of preceding antineoplastic therapy for each patient. BOR is defined as best documented response achieved under NSTT. DCR is defined as proportion of patients who achieved complete response (CR), partial response (PR) or stable disease (SD) as best response; ORR is defined as proportion of patients who achieved either CR or PR as best response. TTR is defined as time from start of treatment to first objective response observed for patients who achieved a CR or PR. DOR is defined as time from documentation of tumor response to disease progression or death from any cause. TTF is defined as time from start of therapy to discontinuation of treatment for any reason, including progression, toxicity, and death.

Patient subgroups based on different substance classes will be analyzed separately. Provided that numbers are sufficient, further subgroups will be analyzed, e.g. by tumor entity, biomarker, intervention. All subgroup analyses will be considered exploratory.

Interim analyses are planned to be conducted after each data collection phase (2020, 2021, 2022) and a final analysis will be conducted at the end of study (2023).

Statistical analyses will be performed using SAS software, version 9.4 or higher and R version 4.0.3 or higher.

## Discussion

Physicians strive to identify the most appropriate and effective cancer treatment for each individual patient. However, reliable evidence for precision oncology approaches to guide physicians in their therapy decisions is still scarce. Thus, there is an urgent need to provide more evidence to determine relevant drug/alteration associations [24].

Only about 5% of cancer patients participate in clinical trials [25], often due to strict inclusion criteria [26]. With the increasing number of molecularly defined patient groups, the overall prevalence of patients with such alterations becomes even smaller, which makes recruitment into trials even more challenging. However, the element of randomization is still a very important technique in clinical research to investigate patient benefits and harms. The SHIVA trial as well as the ongoing international CUPISCO trial (NCT03498521) [27] are strong examples for the feasibility of randomization in precision oncology trials, supporting the conduction of randomized controlled trials in this research field. The goal of these two examples is not to test a specific drug against a standard of care, but to compare the whole approach of testing and recommended treatment against standard of care. Nevertheless, it is also of great importance to understand current practice of precision oncology approaches outside clinical trials. One of the major challenges in the field of precision oncology is the standardization of testing methods, their interpretation and actual implication for clinical decision making. These are research fields that can preferably be investigated in cohort studies with very broad inclusion criteria. In addition, data on effectiveness can easily be explored for hypothesis generation and non-randomized comparative analyses. Thus, establishing a registry study such as INFINITY will provide complementary evidence to the complex field of precision oncology leveraging data that combine patient’s clinical characteristics, the tumor genotype together with the treatment journey and clinical outcomes in real-world.

The non-interventional design of the INFINITY study represents both a limitation, as it precludes causal conclusions on differences between subgroups, and a strength because it allows presentation of real-world data on patients not selected by restrictive inclusion criteria and on test rates in routine care. The retrospective documentation is a potential risk of bias for reporting patients with a better response to NSTT by participating sites. However, this was mitigated by defining certain time periods for documentation. A strength of this study is the enrollment from both hospitals and office-based oncologists in private practices all over Germany, recruiting a large, representative study cohort.

In summary, the INFINITY registry aims to provide real-world data, including the clinical outcomes, on molecular alteration-matched targeted therapies used outside of their approved indication in patients with advanced solid tumors or hematologic malignancies. Thus, the results will describe how precision oncology is currently applied and implemented in routine clinical practice in Germany. Furthermore, the results will complement available evidence on effectiveness of precision oncology in routine clinical practice in general, and of specific drug class/alteration matches used outside their approved indications.

## Data Availability

The datasets used and/or analyzed during the current study are available from the corresponding author on reasonable request.

## Abbreviations

BOR: Best overall response
BRAF: v-raf murine sarcoma viral oncogene homolog B1
BRCA1/2: Breast cancer 1/2
DCR: Disease control rate
dMMR: Mismatch repair deficient
DOR: Duration of response
ECOG: Eastern Cooperative Oncology Group
eCRF: Electronic case report form
ERBB2: Erb-b2 receptor tyrosine kinase 2
FAS: Full analysis set
FDA: Food and Drug Administration
FISH: Fluorescent in situ hybridization
FLT-3: FMS-like tyrosine kinase-3
HER2: Human epidermal growth factor receptor-2
HGVS: Human genome variation society
IHC: Immunohistochemistry
MSI-H: Microsatellite instability-high
MTB: Molecular tumor board
NGS: Next generation sequencing
NSTT: Non-standard targeted therapy
NTRK: Neurotrophic tyrosine receptor kinase
ORR: Overall response rate
OS: Overall survival
RT-PCR: Reverse transcription polymerase chain reaction
PD-L1: Programmed death-ligand 1
PFS: Progression-free survival
RET: Rearranged during transfection
TMB-H: High tumor mutational burden
TTF: Time to treatment failure
TTR: Time to response
